# Optimal Approaches for Measuring Tongue-Pressure Functional Reserve

**DOI:** 10.1155/2013/542909

**Published:** 2013-02-14

**Authors:** Catriona M. Steele

**Affiliations:** Toronto Rehabilitation Institute, University Health Network, 550 University Avenue, Toronto, ON, Canada M5G 2A2

## Abstract

Tongue-palate pressure is a parameter of considerable interest in the field of dysphagia. Maximum isometric tongue-palate pressures (MIPs) decline in healthy aging and in dysphagia. Functional reserve (FR) is the difference between MIPs and swallowing pressures. Reduced FR is thought to constitute a risk for developing functional swallowing impairments. We compare different approaches for calculating FR and recommend an optimal approach. Tongue-palate pressure data were collected from 78 healthy adults (40 < age  40; 38 > 60) during anterior and posterior MIPs, regular (RESS) and effortful (ESS) saliva swallows, and water swallows (4 repetitions per task). Six different measures of reserve were calculated using maximum anterior MIPs or ESS pressures at the top, and mean or maximum RESS or water swallow pressures at the bottom of the range. Correlations with age and MIPs were explored to confirm suitability for measuring FR. The impact of normalization to maximum MIP range was explored. We conclude that an optimal measure of FR involves the comparison of maximum MIP with mean saliva swallowing pressures. This parameter declines with age, but when normalized to an individual's MIP range, the relationship is no longer evident. This suggests that FR does not necessarily decline in healthy aging.

## 1. Introduction

The ability to generate tongue-palate pressure has emerged as a measure of considerable clinical and research interest in the field of dysphagia over the past two decades. Key to this interest is that tongue strength, measured during maximum isometric tongue-palate pressure tasks (MIPs), appears to decline in healthy aging [1–6]. This has been argued to resemble sarcopenia, a degenerative loss of skeletal muscle mass and strength seen in aging in the limb musculature. Reduced tongue strength has also been observed in adults with dysphagia [7–10]. These observations have prompted research into exercise-based methods for tongue strengthening in the hope that greater tongue strength and endurance will lead to improved swallowing function [9, 11, 12].

Functional reserve is the term coined to describe the difference in pressures generated in maximum isometric pressure (MIP) tasks compared to swallowing tasks. Robbins and colleagues were the first to point out that swallowing pressures appear to be preserved in healthy older adults, even in the presence of reduced MIPs. Reductions in functional reserve, due to reductions in MIPs, were argued to have important clinical implications and to place a person at greater risk of developing functional swallowing impairments, particularly in the case of decompensation [2, 13].

Functional reserve describes a tongue-palate pressure range that is bounded at the top by a maximum pressure task and at the bottom by a swallowing task. The swallowing task used to define the bottom of the functional reserve range has varied across studies, leading to some confusion regarding the measure. Furthermore, different instruments have been used across studies, without clear demonstration that measures can be generalized across instruments. We are conducting a program of research exploring the relationship between tongue pressure capacity and swallowing behaviors. We are specifically interested in confirming whether or not limited tongue pressure capacity contributes to constrained variability in swallowing behaviors and whether such constraints are seen as a function of healthy aging. We have recently reported that functional reserve, which we defined as the difference between MIPs and regular effort saliva swallow (RESS) pressures, does not necessarily decline with age [14]. Although overall groupwise trends showed significantly larger functional reserve in individuals aged 20–40 compared to those over the age of 60, we noticed that 18% of our older participant group had excellent functional reserve above the 75th percentile for pooled functional reserve in our sample. Similarly, 13% of our younger group had limited functional reserve, below the 25th% percentile. 

Of particular note is the study of Youmans et al. [5], which involved a functional reserve calculation using bolus swallowing pressures at the bottom of the range, with these pressure measures transformed to a percentage value relative to each participant's MIP range, thereby controlling individual differences in strength. These authors found no significant age-group differences using this transformation, although women were found to use a higher percentage of their MIP range when swallowing boluses compared to men. Youmans and colleagues argued that these results challenged the conventional view that functional reserve declines in healthy aging. Similarly, a previous study by Yeates and colleagues [15] measured a parameter that they called “swallow reserve,” comparing effortful saliva swallows to regular effort saliva swallows, and failed to find age-related differences in this measure. 

These observations led us to question whether functional reserve is indeed a parameter that changes in healthy aging and to explore which elements need to be included in a robust and psychometrically valid measure of functional tongue pressure reserve. We propose that such a parameter should display three characteristics.

- A measure of functional reserve should be sensitive to, and positively correlated with, changes in maximum strength measures (MIPs).
- A measure of functional reserve should be sensitive to changes that occur across the age span, but in order for these to support a hypothesis that functional reserve declines with age, the trend should be visible in a parameter where swallowing pressures are expressed as a percentage of a person's maximum tongue strength, in order to control for individual variations in strength.
- A measure of functional reserve should be one that can be collected easily, without posing a risk of aspiration for the patient.

In this paper, we explore 6 different candidate measures of functional reserve to determine an optimum method for capturing functional reserve in future research and clinical situations.

## 2. Materials and Methods

Tongue pressures at the anterior, medial, and posterior palate were collected from 78 healthy consenting adults in two sex-balanced age groups. The younger participant group ranged in age from 18 to 39 years, with a mean age of 27 years. The older participant group ranged in age from 60 to 87, with a mean age of 70 years. The protocol was approved by the local institutional research ethics board. Exclusion criteria included participant-reported history of type I diabetes, chronic sinusitis, taste disturbance, or any swallowing, motor speech, gastroesophageal, or neurological difficulties. During intake, a brief oral mechanism examination and a water swallow screening were performed by a licensed speech-language pathologist to confirm eligibility to participate.

### 2.1. Data Collection

We used the 3-bulb tongue array of the KayPentax Swallowing Signals Lab to collect tongue pressures, with the bulbs spaced 8 mm apart and adhered to the palate in midline using Stomahesive (Convatec, St-Laurent, Quebec, Canada). The anterior bulb was positioned on the alveolar ridge, immediately behind the upper incisors. Pressure signals were sampled at 250 Hz with the range calibrated to record an upper amplitude limit of 750 mmHg. Five different pressure tasks were included, with each performed in a block of 4 task repetitions. The protocol commenced with the maximum anterior isometric pressure task (AMAX) and then proceeded to one of four randomly assigned sequences for the collection of regular effort saliva swallows (RESS), effortful saliva swallows (ESS), maximum posterior isometric pressures (PMAX), and discrete water swallows (i.e., single sips of ~8–10 mL taken from a cup). The PMAX data have been reported elsewhere and are not used for the calculations reported in this paper.

### 2.2. Data Processing

**Figure 1:**
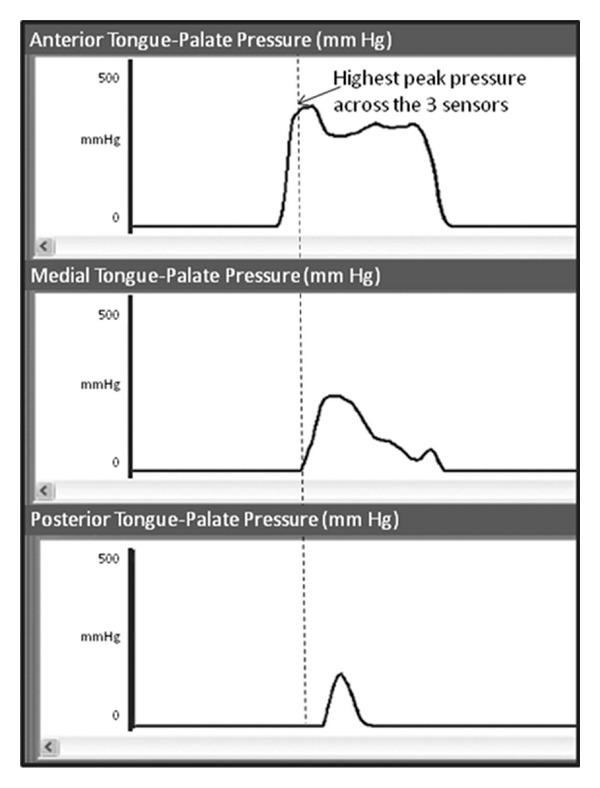
Illustration of tongue-palate pressure waveforms on the KayPentax Digital Swallow Workstation. The highest peak pressure (in mm Hg) across the anterior, medial, and posterior sensors was used as the value of peak pressure for each task.

Anterior, medial, and posterior palate pressure waveforms were displayed on a computer monitor. The onset, peak, and offset of each pressure event were indexed by a trained research assistant and pressure amplitudes at each of these timepoints were recorded. Pressure amplitude (in mm Hg) was calculated as the amplitude difference (in mm Hg) between the highest peak pressure amplitude and the lowest baseline pressure amplitude (usually zero mm Hg) seen across all three pressure waveforms (anterior, medial, and posterior) for a given task repetition.  Figure 1 provides an illustration of this parameter. 

### 2.3. Analysis

Six different equations were calculated as possible measures of functional reserve as follows.

- Maximum peak pressure on the AMAX task minus maximum peak pressure on the water swallowing task.
- Maximum peak pressure on the AMAX task minus mean peak pressure on the water swallowing task.
- Maximum peak pressure on the AMAX task minus maximum peak pressure on the RESS task.
- Maximum peak pressure on the AMAX task minus mean peak pressure on the RESS task.
- Maximum peak pressure on the ESS task minus maximum peak pressure on the RESS task.
- Maximum peak pressure on the ESS task minus mean peak pressure on the RESS task.

Scatter plots were prepared in IBM SPSS 19.0, plotting each measure of functional reserve, first as a function of maximum isometric pressure (maximum peak pressure on the AMAX task) and second as a function of age. An *a priori* criterion for correlations of interest was established as a Pearson's correlation of *R* = 0.4. Finally, RESS and water swallow pressure values were transformed to a percentage value of each participant's maximum isometric pressures, and scatter plots and correlations were again explored using these transformed data.

## 3. Results and Discussion

Table 1 provides descriptive statistics for the observed Pearson's correlations between each candidate measure of functional reserve: (a) maximum isometric pressure and (b) age. 

**Figure 2:**
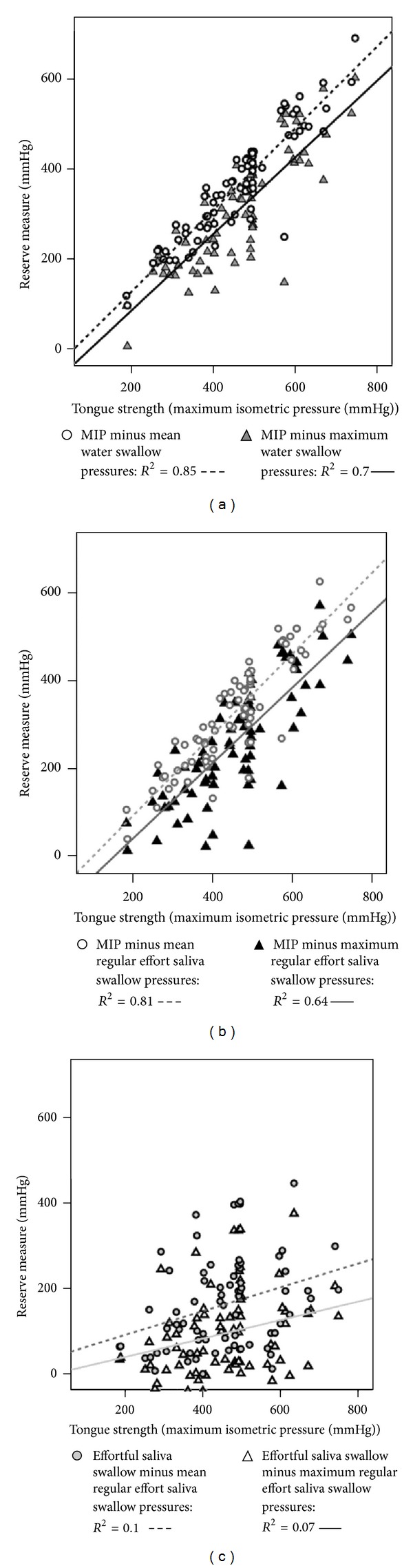
(a) Scatter plot showing the relationship between functional reserve measures comparing maximum isometric pressure (MIP) to water swallowing pressures and tongue strength, as measured by MIP. (b) Scatter plot showing the relationship between functional reserve measures comparing maximum isometric pressure (MIP) to regular effort saliva swallowing pressures and tongue strength, as measured by MIP. (c) Scatter plot showing the relationship between functional reserve measures comparing maximum effortful saliva swallowing pressures to regular effort saliva swallowing pressures and tongue strength, as measured by MIP.

As shown in Figure 2(a), when the traditional approach for measuring functional reserve is used (MIP minus water swallowing pressures), there are strong correlations with MIP, regardless of whether mean (*R* ^2^ = 0.85) or maximum (*R* ^2^ = 0.70) water swallowing pressure values are used at the bottom of the functional reserve range. Similarly, as shown in Figure 2(b), strong correlations are also seen when either mean (*R* ^2^ = 0.81) or maximum (*R* ^2^ = 0.64) saliva swallow pressures are used at the bottom of the functional reserve range. By contrast, as shown in Figure 2(c), measures of swallow reserve, in which effortful saliva swallows are used at the top of the functional reserve range, do not correlate strongly with MIP when either mean (*R* ^2^ = 0.10) or maximum (*R* ^2^ = 0.07) saliva swallows are used at the bottom of the range. This suggests that swallow reserve, as proposed by Yeates and colleagues [15], is not sensitive to differences in tongue pressure capacity and is therefore not a good measure of functional reserve.

**Figure 3:**
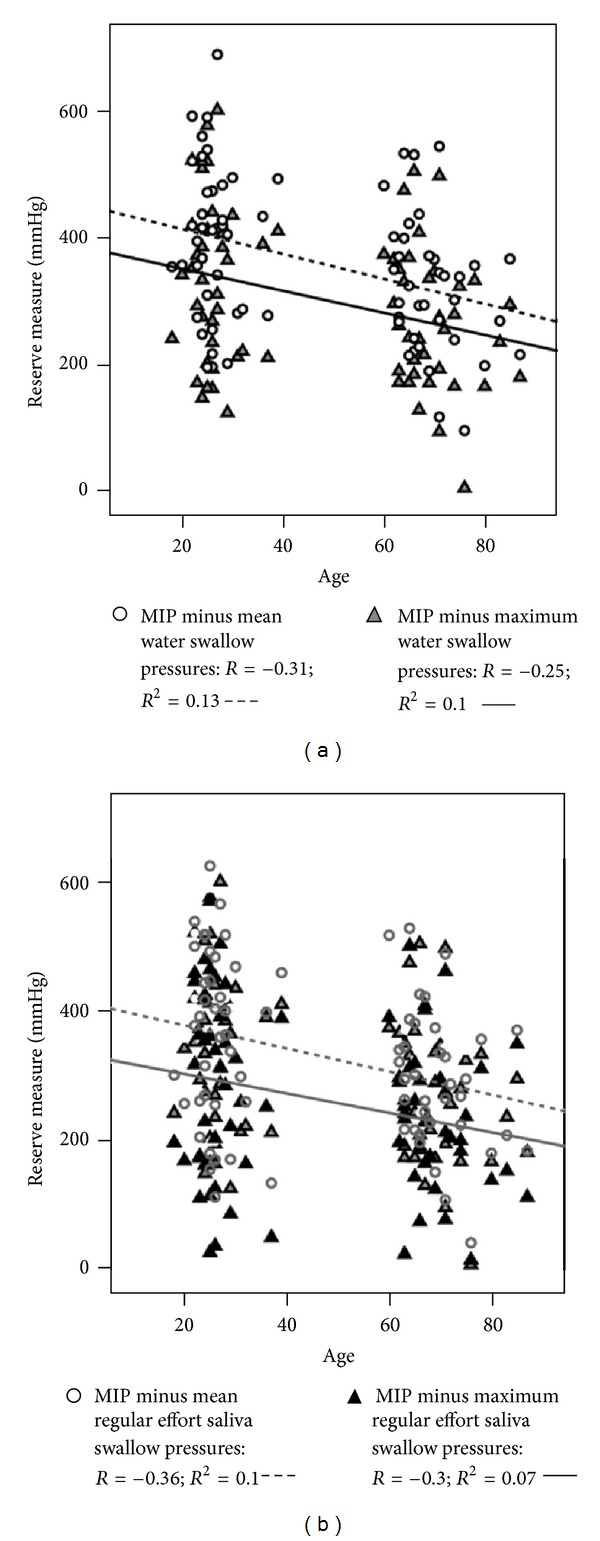
(a) Scatter plot showing the relationship between functional reserve measures comparing maximum isometric pressure (MIP) to water swallowing pressures and age. (b) Scatter plot showing the relationship between functional reserve measures comparing maximum isometric pressure (MIP) to regular effort saliva swallowing pressures and age.

With respect to the relationship between functional reserve measures and age, Figure 3(a) shows weak but statistically significant negative correlations with age for functional reserve measures comparing MIP to mean (*R* = −0.31, *R* ^2^ = 0.13) or maximum (*R* = −0.25, *R* ^2^ = 0.10) water swallowing pressures. Similarly, as shown in Figure 3(b), weak but statistically significant correlations with age are seen when either mean (*R* = −0.36, *R* ^2^ = 0.10) or maximum (*R* = −0.30, *R* ^2^ = 0.07) saliva swallow pressures are used at the bottom of the functional reserve range. Age correlations with swallow reserve were not explored given the earlier result showing a lack of sensitivity to changes in strength.

**Figure 4:**
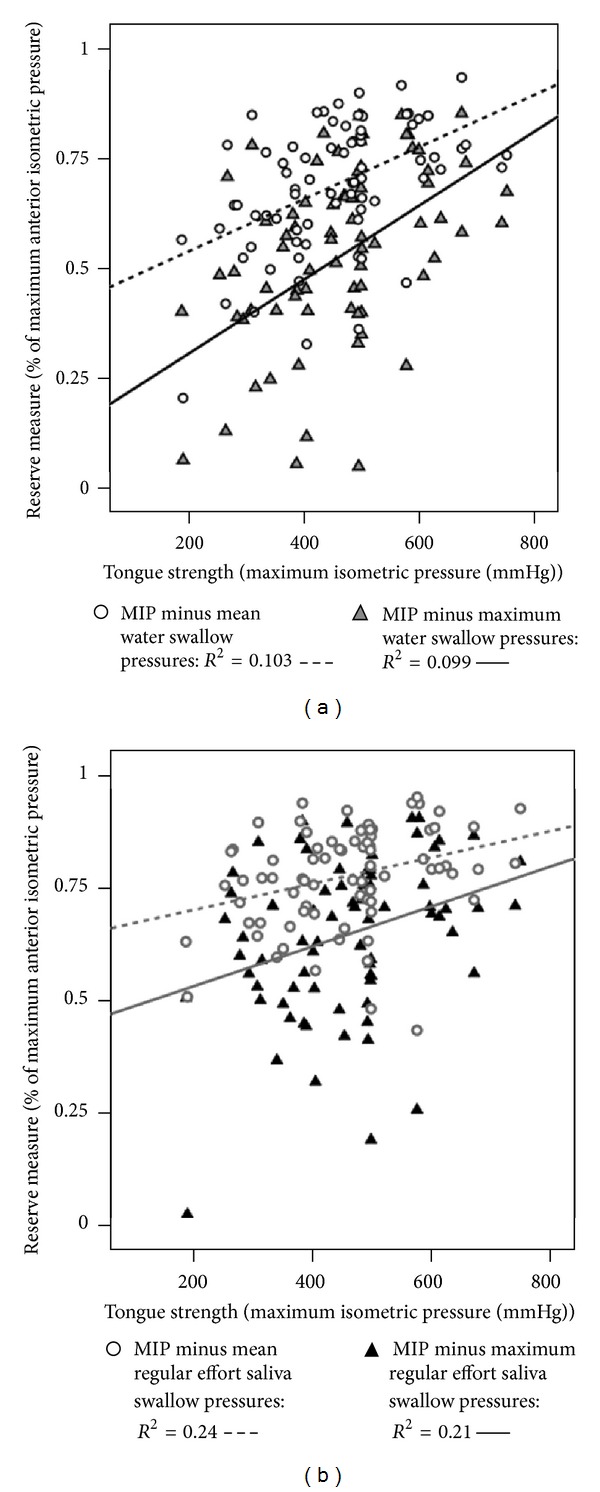
(a) Scatter plot showing the relationship between functional reserve measures comparing maximum isometric pressure (MIP) to water swallowing pressures and tongue strength, as measured by MIP, with swallow pressures expressed as a percentage of MIP. (b) Scatter plot showing the relationship between functional reserve measures comparing maximum isometric pressure (MIP) to regular effort saliva swallowing pressures and tongue strength, as measured by MIP, with swallow pressures expressed as a percentage of MIP.

**Figure 5:**
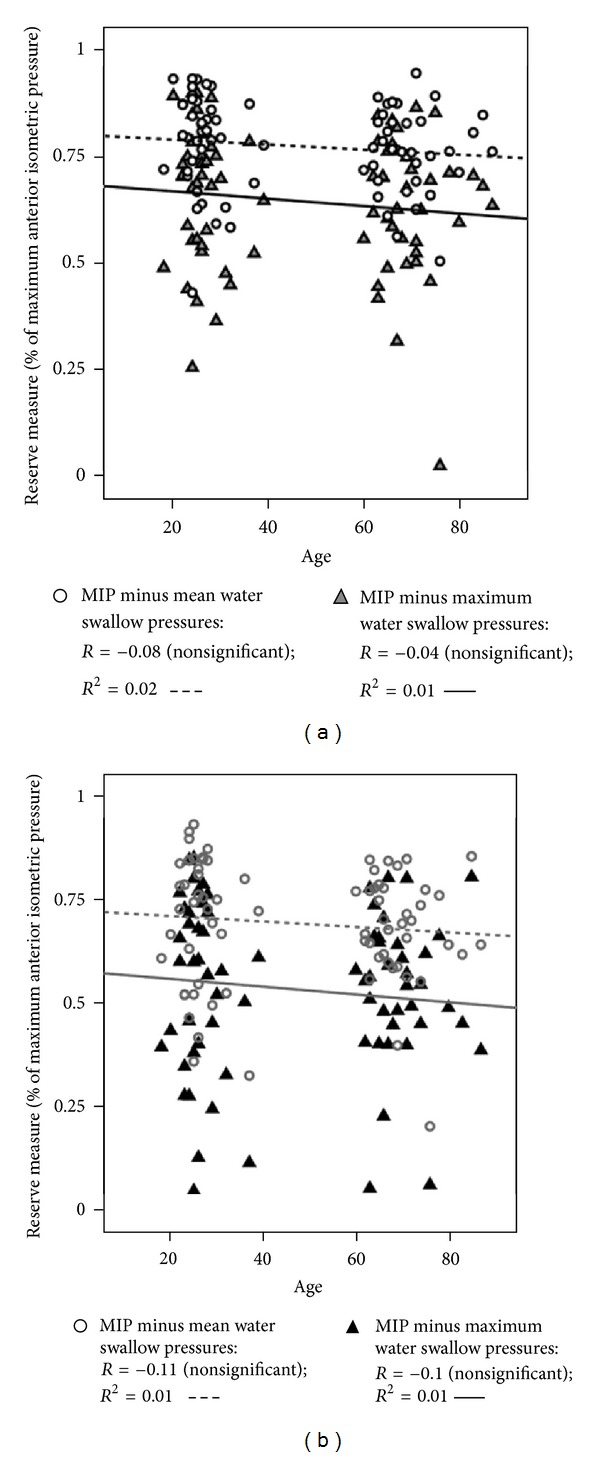
(a) Scatter plot showing the relationship between functional reserve measures comparing maximum isometric pressure (MIP) to water swallowing pressures and age, with swallow pressures expressed as a percentage of MIP. (b) Scatter plot showing the relationship between functional reserve measures comparing maximum isometric pressure (MIP) to regular effort saliva swallowing pressures and age, with swallow pressures expressed as a percentage of MIP.

Finally, we explored what happened to the previously observed correlations when we transformed our measures of swallowing pressure into values normalized to a percentage of each participant's maximum strength measure. As shown in Figure 4(a), strong correlations with MIP are retained with this transformation when saliva swallows are used at the bottom of the functional reserve range. With water swallows, the correlations fall just below the *a priori* criterion of *R* = 0.4, but remain statistically significant (Figure 4(b)). With respect to correlations with age, if a correlation age remains significant after this transformation, we would be able to conclude that functional reserve declines as a function of age, rather than being primarily driven by a person's strength. This, however, was not the case. Figures 5(a) and 5(b) show that the previously observed weak correlations with age are negated by the transformation. No statistically significant correlations with age are seen, regardless of the equation used to calculate functional reserve. This result confirms the previous result reported by Youmans and colleagues [5], suggesting that the appearance of an age-related decline in functional reserve may be an artifact attributable to variability in MIP measures across participants. 

## 4. Conclusions

We conclude that optimally robust measures of functional reserve are those comparing maximum tongue strength (MIPs) to mean water or saliva swallowing pressures. Of these two options, saliva swallows involve less risk of aspiration during data collection. The results of the analysis in this study suggest that it would be reasonably straight forward for clinicians to routinely incorporate the measurement of tongue pressure functional reserve into swallowing assessment, using a short series of MIPs and saliva swallows. 

We recommend that functional reserve measures should use a data transformation to account for individual differences in maximum tongue strength measures, by expressing swallowing pressures as a percent of a person's MIP. Using this transformation, functional reserve measures comparing MIP to either mean or maximum saliva swallow pressure retain statistically significant strong correlations with strength. However, this transformation has the effect of negating apparent age-related differences in functional reserve. 

When normalized to control individual variations in strength, our data concur with those of Youmans and colleagues [5] in showing that functional reserve does not decline as a function of healthy aging. This finding strongly suggests that older adults should not be assumed to have tongue weakness and reduced functional reserve simply as a function of age. Indeed, our data suggest that the distribution of reduced functional reserve is not age dependent and that individuals with limited functional reserve may be found in the younger population. Certainly, prior studies point to limited functional reserve being a characteristic of dysphagia. The consequences of functional reserve for swallowing function and the ability to benefit from commonly used interventions such as texture modification require further elucidation in future studies. Similarly, the benefits of tongue pressure resistance training for building functional reserve and restoring more normal swallowing function are still to be clearly demonstrated.

## Figures and Tables

**Table 1:** Pearson's correlations between different measures of functional reserve: maximum isometric pressure and age.

Functional reserve measure	Unit of measurement	Maximum isometric pressure (MIP)		Age	
		Pearson's correlation	Significance (2-tailed)	Pearson's correlation	Significance (2-tailed)
MIP minus maximum water swallow pressures	mm Hg	**.814**	.000	−.304	.007
	% MIP	.314	.005	−.104	.368
MIP minus mean water swallow pressures	mm Hg	**.910**	.000	−.359	.001
	% MIP	.320	.005	−.107	.354
MIP minus maximum regular effort saliva swallow pressures	mm Hg	**.796**	.000	−.250	.031
	% MIP	**.453**	.000	−.042	.716
MIP minus mean regular effort saliva swallow pressures	mm Hg	**.899**	.000	−.312	.006
	% MIP	**.493**	.000	−.078	.500
Maximum effortful minus maximum regular effort saliva swallow pressures	mm Hg	.285	.013	n/a	n/a
Maximum effortful minus mean regular effort saliva swallow pressures	mm Hg	.325	.004	n/a	n/a

Correlations with *R* > 0.4 are shown in bold.
